# The role of depressive symptoms and social support in the association of internet addiction with non-suicidal self-injury among adolescents: a cohort study in China

**DOI:** 10.1186/s12888-023-04754-4

**Published:** 2023-05-09

**Authors:** Ying Ma, Yanqi Li, Xinyi Xie, Yi Zhang, Brooke A. Ammerman, Stephen P Lewis, Ruoling Chen, Yizhen Yu, Fenghua Li, Jie Tang

**Affiliations:** 1 Department of Child Healthcare, Guangzhou Women and Children’s Medical Center, Guangzhou Medical University, Guangzhou, P.R. China; 2 Department of Preventive Medicine, School of Public Health, Guangzhou Medical University, Room 507, Block 2, Jinxiu Road, Xinzao, Panyu District, Guangzhou, 511436 P.R. China; 3 Department of Psychology, University of Notre Dame, Notre Dame, IN USA; 4 Department of Psychology, University of Guelph, Guelph, ON Canada; 5 Faculty of Education, Health and Wellbeing, University of Wolverhampton, Wolverhampton, UK; 6 Department of Maternal and Child Healthcare, School of Public Health, Tongji Medical College, Huazhong University of Science and Technology, Wuhan, P.R. China; 7 Zhongshan Health Care Center for Primary and Secondary Schools, Zhongshan City, P.R. China

**Keywords:** Internet addiction, Non-suicidal self-injury, Depressive symptoms, Social support, Cohort study, Adolescent

## Abstract

**Background:**

Both internet addiction (IA) and non-suicidal self-injury (NSSI) are major public health concerns among adolescents, however, the association between IA and NSSI was not well understood. We aimed to investigate the association between IA and NSSI within a cohort study, and explore the mediated effect of depressive symptoms and the moderating effect of social support in the association.

**Methods:**

A total of 1530 adolescents aged 11–14 years who completed both the baseline (T1) and 14-month follow-up (T2) survey of the Chinese Adolescent Health Growth Cohort were included for the current analysis. IA, NSSI, depressive symptoms and social support were measured at T1; depressive symptoms and NSSI were measured again at T2. Structural equation models were employed to estimate the mediated effect of depressive symptoms and the moderating effect of social support in the association between IA and NSSI at T2.

**Results:**

IA was independently associated with an increased risk of NSSI at T2, with the total effect of 0.113 (95%CI 0.055–0.174). Depressive symptoms mediated the association between IA and NSSI at T2, and social support moderated the indirect but not the direct effect of IA on NSSI at T2. Sex differences were found on the mediated effect of depressive symptoms and the moderated mediation effect of social support.

**Conclusions:**

Interventions that target adolescents’ NSSI who also struggle with IA may need to focus on reducing depressive symptoms and elevating social support.

**Supplementary Information:**

The online version contains supplementary material available at 10.1186/s12888-023-04754-4.

## Background

Over the past few decades, non-suicidal self-injury (NSSI) has become a major public health concern among children and adolescents [1, 2]. Data from the latest survey of the World Mental Health International College Students (WMH-ICS) indicated the lifetime and 12-month prevalence of NSSI were 17.7% and 8.4%, respectively, among first year college students [3]. In China, the prevalence of NSSI among middle and high school students ranged from 6.4% to 35.6% [4]. NSSI is associated and comorbid with several mental disorders, such as major depression, bipolar disorder, substance and alcohol abuse [3–5]. What is more, NSSI increases the risk of both suicide and other causes of mortality [6]. Given the high prevalence and serious consequences of NSSI, it is critical to further understand its risk and protective factors.

Internet addiction (IA), usually defined as the inability to control one’s internet use and causes marked distress and/or functional impairment [7], is also highly prevalent among adolescents worldwide [8, 9]. A recent study estimated that the prevalence of IA among children and adolescents in Macau and mainland China was 23.7% [10]. Our previous study [11] and other studies [12, 13] have suggested that IA may be associated with NSSI among adolescents. However, it is uncertain whether exposure to IA is prospectively associated with an increased risk of NSSI among adolescents given the lack of evidence from longitudinal studies in the field [13].

In addition to IA, studies on clinical and general populations have suggested that depressive symptoms may be an independent risk factor of NSSI [14, 15]. Longitudinal studies have also suggested that compulsive internet use may precede the development of emotional dysregulation [16], and depressive symptoms are one of the most frequent consequences of IA [17]. It seems that depressive symptoms might mediate the association between IA and NSSI. However, only a limited amount of research has specifically investigated the mediated role of depressive symptoms in the association between IA and NSSI. Nevertheless, several previous studies have suggested that depression may mediate the association between interpersonal stressors and NSSI among adolescents [18].

Additionally, according to the stress-buffering hypothesis [19], social support moderates the association between stress and health outcomes. Previous studies have suggested that a high level of social support could relieve depressive symptoms and protect against the onset of major depression [20, 21], as well as buffer the effect of life stress on NSSI [22]. Thus, social support may exert moderating effects on both the associations between IA and depressive symptoms, and between IA and NSSI. However, little research has yet examined the moderating role of social support in these associations. Further research on the underlying role of depressive symptoms in the association between IA and NSSI, along with whether social support can moderate these associations, is necessary. If these possible relations are supported by evidence, it would help scholars and educators to better understand the nature of the association between IA and NSSI among children and adolescents.

Using a longitudinal design, the present study aimed to explore the role of depressive symptoms and social support in the association of IA and NSSI. Given that early adolescence has a high rate of NSSI and this age range represents a typical onset time of NSSI [23], we investigated a school-based sample of early adolescence to explore these relations. Specifically, we constructed a mediation model and moderated-mediation model and proposed the following hypotheses: (1) IA would be prospectively associated with NSSI among adolescents; (2) depressive symptoms would mediate the association between IA and NSSI; and (3) social support would moderate the association between IA and NSSI, and the association between IA and depressive symptoms in the mediation model of the association between IA and NSSI.

## Methods

### Study participants

This study used data from the Chinese Adolescent Health Growth Cohort (CAHGC) study (register number: CCC2022061901, http://chinacohort.bjmu.edu.cn), which aimed to investigate the development trajectory and influencing factors of risk behaviors (mainly on NSSI and aggression) among adolescents. The design, procedure, and implementation were described elsewhere [24]. In brief, using a cluster random sampling method, a representative sample of 1844 students at grade 7 from 11 middle schools across three areas (Qidong County in Hunan Province, Guangming District in Shenzhen City, Zhongshan City in Guangdong Province) were enrolled to participate in the baseline survey (T1). Participants then completed a follow-up survey that was undertaken at 14 months after baseline (T2, N = 1758). Overall, there were 1543 participants who completed both T1 and T2 surveys. After excluding 13 participants who did not complete the assessment of IA at T1 and/or NSSI at T2, the final sample included 1530 participants. No significant differences were found in the variables of interests (i.e., IA, depressive symptoms, social support and NSSI) or other demographic variables (such as age and sex) between adolescents who participated in all assessments and these who did not. The study followed the Strengthening the Reporting of Observational Studies in Epidemiology (STROBE) guideline and received ethics clearance from the University (NO.2021010002). In each round of data collection, the parents/guardians provided written informed consent that was in a manner consistent with the Declaration of Helsinki. Participants in the study received the equivalent of RMB 150 ($25) in medical examination services after the baseline survey.

### Instruments

**IA**. At T1, IA was assessed by using the Young Internet Addiction Test (IAT) [25]. The IAT comprises 20 items using a 5-point Likert scale: 1, rarely; 2, occasionally; 3, frequently; 4, often; 5, always; thus, total scores of the IAT range from 20 to 100. Three types of internet user groups were identified based on the original cutoff points proposed by Young [26], namely “average online users” (20–49 points), “moderate IA” (50–79 points), and “severe IA” (80–100 points) [26]. The IAT has been demonstrated to have acceptable internal consistency, with a Cronbach α coefficient of 0.93 in a previous study [11] and 0.91 in the present study sample.

**Depressive symptoms**. At T1 and T2, a Chinese version of the Center for Epidemiological Studies Depression Scale (CES-D) was used to measure depressive symptoms [27], which consists of 9 items using 4-point Likert responses: 0 = never true; 1 = rarely true; 2 = often true; and 3 = always true; thus, the total scores on CES-D range from 0 to 27. Higher scores indicate greater risk of depression due to more severe depressive symptoms. Participants were divided into three groups based on the validated standard cutoff criteria, including ‘no depressive symptoms’ (0–9 points), ‘moderate depressive symptoms’ (10–16 points), and ‘severe depressive symptoms’ (17–27 points). The Cronbach α coefficient of the scale in the present study was 0.83.

**Social support**. At T1, social support was measured by the 17-item Adolescents Social Support Scale [28], which has 5-point Likert responses for each item (1 = strongly; 2 = agree; 3 = neutral; 4 = somewhat disagree; 5 = strongly disagree). The total scores of the scale ranged from 17 to 85, where higher scores of the scale indicate higher level of social support. The Cronbach α coefficient of the scale in the present study was 0.96.

**NSSI**. At T1 and T2, the Chinese version of the Functional Assessment of Self-mutilation was used to assess the frequencies of eight different forms of NSSI (hitting, head banging, stabbing, pinching, scratching, biting, burning, and cutting) during the past 12 months [29]. To distinguish between NSSI and suicidal behaviors, participants were also asked whether any of those behaviors carried suicidal intent. Similar to our previous studies [11, 29], NSSI was dichotomized (frequency of five or more acts in the past year = yes; versus fewer than five = no) for analysis. The Cronbach’s alpha of this scale in the present study was α = 0.98.

Co-variables. At T1, we used a custom-designed questionnaire to collect demographic characteristics, familial, and parenting variables, including age, sex (male or female), ethnicity (Han or others, meaning, every category of ethnicity except Han), single-child family (yes or no), family structure (core/joint family, single parent/blended family, cross-generation family), education level of the main caregiver (middle school or below, high school or technical school, college or above), family income (< $200/month, $200–900/month, or > $900/month) [29], family history of psychiatric diseases (yes or no) and perceived academic pressure (high, average, or low). Parenting style was measured by a single question of “what kind of parenting style do you think that your main caregivers treat you?” and four options were given (strict, pampered, neglect or frequently changing, or open-minded). A previous study had shown that the test-retest reliability of the questionnaire was α = 0.83 [29].

### Statistical analysis

Frequencies and proportions for categorical variables or mean (SD) for continuous variables were used to describe participant characteristics and NSSI at T2 by study variables. χ^2^ tests or 2-tailed, unpaired *t* tests were used to compare the distribution between participants with or without a history of NSSI at T2 according to studied variables. Pairwise correlation analysis of measurements (IA and social support [T1], depressive symptoms and NSSI [T1 and T2]) was used to test the associations among the variables. We employed log-binominal models based on generalized estimating equations to estimate the adjusted risk ratio (aRR) and 95% confidence interval (CI) of NSSI at T2 for participants who were IA at T1. In the adjusted model, we adjusted for age, sex, ethnicity, study site, family structure, single-child family, education level of main caregiver, parenting style, family income, family history of psychiatric disease, perceived academic pressure, NSSI and depressive symptom at T1. We also conducted subgroup analysis to examine whether the association between IA and NSSI at T2 differed by sex. Significance level was set at *P* < 0.05 and all tests were 2-sided. Statistical analyses were conducted using IBM SPSS Statistics, version 26.0.

We performed a set of structural equation models (SEM) to estimate the mediated effect of depressive symptoms in the association between IA and NSSI at T2, and the moderating effects of social support in the direct and the indirect association between IA and NSSI at T2. Supplementary Figure 1 shows the theoretical framework underlying our mediation analysis and moderated-mediation analysis. In all modelling analyses, both unadjusted and adjusted effects were estimated. In the adjusted model, we adjusted the confounders as we did in the log-binominal models. In the moderated mediation analysis, simple slope analyses and conditional indirect effect tests (1 SD above and below the mean of the moderator) were performed when an interaction effect of IA and social support was detected. We also preformed subgroup analysis to examine whether the mediated or moderated mediation effects differed by sex. All SEM analyses were performed using Mplus 8.0.

## Results

### Demographic characteristic

We included 1530 participants (853 males [55.8%] and 677 females [44.2%]) in the current analysis. At T1, the age of the participants ranged from 11 to 14 years old (mean [SD] age, 12.9 [0.6] years). More than a half (957 [62.5%]) were from Zhongshan City, 1482 (96.9%) were Han ethnicity, 269 (17.6%) were a single child, and 107 (7.0%) were from single parent or blended family. Additional characteristics are summarized in Table 1.

**Table 1:** Characteristics of participants according to NSSI at T2

Characteristic	Total(n = 1530)	NSSI at T2	
		No (n = 1331)	Yes (n = 199)
**Age** (**T1)** (M, SD) ^*^	12.9 ± 0.6	12.9 ± 0.6	12.8 ± 0.6
**Sex** (n, %) ^*^			
Males	853(55.8)	776(57.6)	87(43.7)
Females	677(44.2)	565(42.4)	112(56.3)
**Regional area** (n, %) ^*^			
Qidong County	383(25.0)	310(23.3)	73(36.7)
Guangming District	190(12.4)	173(13.0)	17(8.5)
Zhongshan City	957(62.5)	848(63.7)	109(54.8)
**Ethnicity** (n, %)			
Han	1482(96.9)	1287(96.7)	195(98.0)
Others	48(3.1)	44(3.3)	4(2.0)
**Single child** (n, %)			
No	1261(82.4)	1084(81.4)	177(88.9)
Yes	269(17.6)	247(18.6)	22(11.1)
**Family structure** (n, %) *			
Core/joint family	1333(87.1)	1175(88.3)	158(79.4)
Single parent/blended family	107(7.0)	88(6.6)	19(9.5)
Cross-generation family	90(5.9)	68(5.1)	22(11.1)
**Family history of psychiatric diseases** (n, %) *			
No	1510(98.7)	1317(98.9)	193(97.7)
Yes	20(1.3)	14(1.1)	6(3.0)
**Parenting style** (n, %) *			
Strict	500(32.7)	447(33.6)	53(26.6)
Open minded	783(51.2)	695(51.4)	88(44.2)
Pamper	54(3.5)	42(3.2)	12(6.0)
Neglect/rude/frequently changing	193(12.6)	147(11.0)	46(23.1)
**Monthly household income per capita** (n, %)			
≤ 1999 RMB	60(3.9)	47(4.13.5)	13(6.5)
2000–6999 RMB	910(59.5)	791(59.4)	119(59.8)
≥7000 RMB	560(36.6)	493(37.0)	67(33.7)
**Education of main caregiver** (n, %)			
Junior and below	914(59.7)	795(59.7)	119(59.8)
Senior or technical school	360(23.5)	307(23.1)	53(26.6)
College and above	256(16.7)	229(17.2)	27(13.6)
**Perceived academic pressure** (n, %) *			
High	706(46.1)	584(43.9)	122(61.3)
General	773(50.5)	704(52.9)	69(34.7)
low	51(3.3)	43(3.2)	8(4.0)
**Depressive symptoms (T1) (n, %)** ^*^			
None	1234(80.7)	1132(85.0)	102(51.3)
Moderate	230(15.0)	164(12.3)	66(33.2)
Severe	66(4.3)	35(2.6)	31(15.6)
**IA (T1) (n, %)** ^*^			
None	1015(66.3)	934(70.2)	81(70.2)
Moderate	461(30.1)	364(27.3)	97(48.7)
Severe	54(3.5)	33(2.5)	21(10.6)
**NSSI(T1) (n, %)** ^*^			
No	1355(88.6)	1224(92.0)	131(65.8)
Yes	175(11.4)	107(8.0)	68(34.2)
**Depressive symptoms(T2) (n, %)** ^*^			
None	1243(81.2)	1135(85.3)	108(81.2)
Moderate	235(15.4)	169(12.7)	66(33.2)
Severe	52(3.4)	27(2.0)	25(12.6)
**Social support(T1)** (M, SD) ^*^	67.0 ± 13.4	68.2 ± 12.8	59.2 ± 14.5

*P < 0.05

Overall, 461 participants met the criteria of moderate IA, and 54 participants met the criteria of severe IA at T1. The prevalence of moderate IA and severe IA were 30.1% and 3.5%, respectively, and no sex differences were found (χ^2^ = 2.281, *P* = 0.320). Participants who were moderate IA or severe IA were likely to have moderate or severe depressive symptoms at T1 (χ^2^ = 19.773, *P* < 0.001). The prevalence of NSSI was 11.4% at T1 and 13.0% at T2, respectively. Univariate analysis on influence factors of NSSI at T2 is also presented in Table 1. Participants who were female, from single parent or blended family, experienced neglect/rude/frequently changing parenting practices, perceived high academic pressure, had moderate or severe IA, had moderate or severe depressive symptoms and engaged NSSI at T1 were more likely to engage in NSSI at T2 (Table 1).

### The independent effect of IA on NSSI at T2

After testing the normality of the independent, dependent variable, mediation and moderating variables, Spearman correlation analyses were performed; results are presented in Supplementary Table 1. The unadjusted RR and adjusted RR of IA for NSSI at T2 were presented in Table 2. After adjusted for the confounders, IA was associated with an increased risk of NSSI at T2. Compared to those who were not IA, participants who were moderate and severe IA had greater aRR of NSSI, the aRR for moderate IA was 2.25 (95%CI: 1.59–3.19); for severe IA was 2.39 (95%CI:1.17–3.08). Subgroup analysis showed that the independent effect of IA at T1 on NSSI at T2 not differed by sex (Supplementary Table 2).

**Table 2:** Association between IA at T1 and NSSI at T2

	NSSI at T2 (N, %)	Unadjusted RR (95%CI)	P		Adjusted ^a^ RR (95%CI)	P
No IA	81 (8.0)	Ref = 1			Ref = 1	
Moderate IA	97 (21.0)	2.64 (2.01–3.47)	< 0.001		2.25 (1.59–3.19)	< 0.001
Severe IA	21 (39.8)	4.87 (3.29–7.23)	< 0.001		2.39 (1.17–3.08)	0.016

a. Adjusted for sex, age, ethnicity, regional areas, family structure, single child, family history of psychiatric disease, parenting style, monthly household income per capita, perceived academic pressure, education of main caregiver, baseline NSSI and baseline depressive symptoms

### The indirect effect of IA on NSSI at T2 mediated through depressive symptoms

**Fig. 1:**
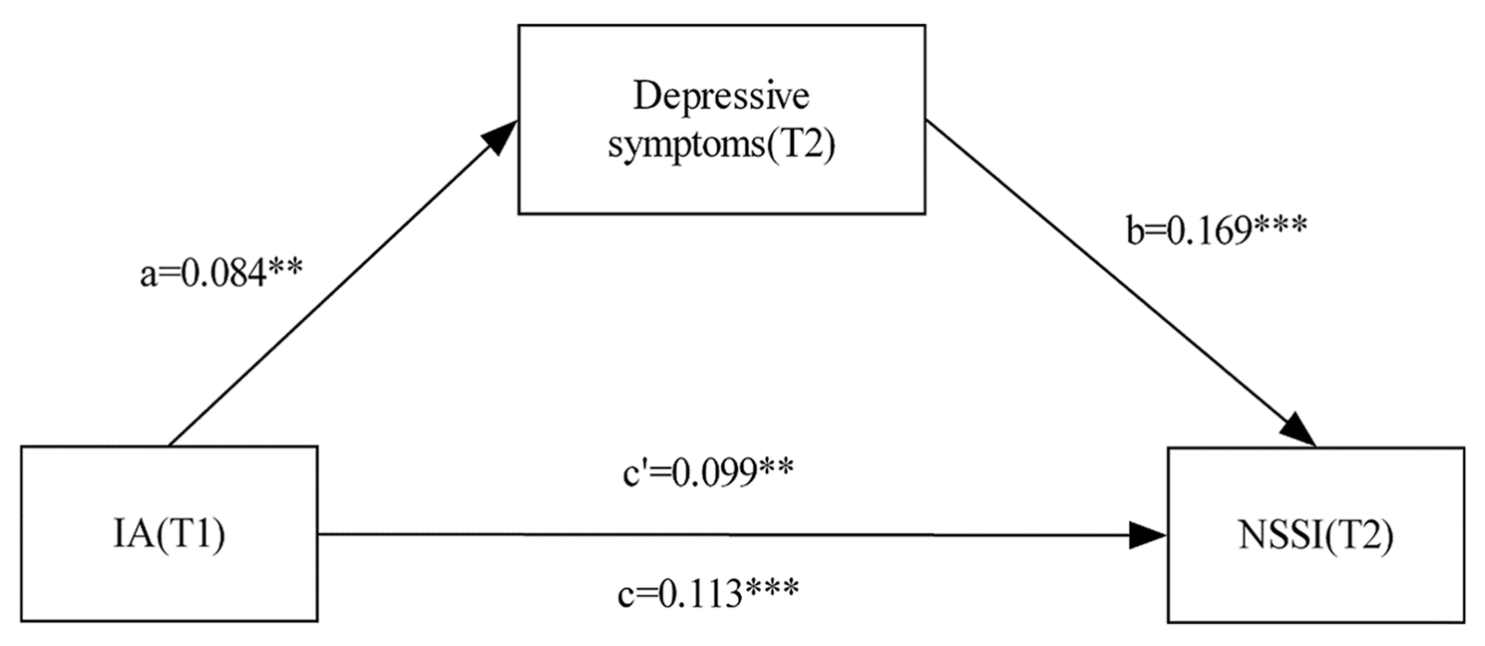
Structural equation modeling depicting direct effects of IA, depressive symptoms on NSSI. Adjusted β coefficients are presented. The model adjusted for sex, age, ethnicity, regional areas, family structure, single child, family history of psychiatric disease, parenting style, monthly household income per capita, perceived academic pressure, education of main caregiver, baseline NSSI and baseline depressive symptoms.^**^p < 0.01,^***^p < 0.001

Figure 1 shows the results of the mediation analyses. After adjusting for potential confounders, the total effect of IA at T1 on NSSI at T2 was significant (standard β = 0.113, 95%CI: 0.055–0.174). The direct effect of IA at T1 on NSSI at T2 was 0.099 (95%CI: 0.042–0.161) and depressive symptoms at T2 on NSSI at T2 was 0.169 (95%CI: 0.100-0.239). The indirect effect of IA on NSSI at T2 mediated through depressive symptoms was 0.014 (95%CI:0.004–0.029). The mediation proportion was 12.4% (95%CI: 7.3-16.7%) (Table 3). Goodness-of-fit indices (i.e., CFI = 1.000, TLI = 1.000, RMSEA < 0.001, SRMR < 0.001) indicated satisfactory fit of the model. Subgroup mediation analysis demonstrated that the total and direct effects of IA at T1 on NSSI at T2 among both males and females were significant (all *P* < 0.001). However, the significant indirect effect of IA at T1 on NSSI at T2 mediated through depressive symptoms was only found for females, with the mediation proportion of 19.0% (95%CI: 15.4-23.8%) (See Supplementary Table 3).

**Table 3:** Mediating effect of depressive symptoms between IA(T1) and NSSI(T2)

Variables	Model 1 ^a^			Model 2 ^b^	
	Standard β (95% CI)	*P*		Standard β (95% CI)	*P*
IA(T1)→Depressive symptoms(T2)	0.200(0.147 to 0.258)	< 0.001		0.084(0.027 to 0.144)	0.005
Depressive symptoms(T2)→NSSI(T2)	0.249(0.181 to 0.314)	< 0.001		0.169(0.100 to 0.239)	< 0.001
IA(T1)→NSSI(T2)	0.179(0.120 to 0.240)	< 0.001		0.099(0.042 to 0.161)	0.001
Standardized effect					
Indirect	0.050(0.032 to 0.072)	< 0.001		0.014(0.004 to 0.029)	0.021
Total	0.229(0.171 to 0.286)	< 0.001		0.113(0.055 to 0.174)	< 0.001
Mediating ratio (%)	21.8(18.7 to 25.2)	--		12.4(7.3 to 16.7)	--

a. Unadjusted

b. Adjusted for sex, age, ethnicity, regional areas, family structure, single child, family history of psychiatric disease, parenting style, monthly household income per capita, perceived academic pressure, education of main caregiver, baseline NSSI and baseline depressive symptoms

### The moderating effect of social support on the mediation path and the direct path in the association between IA and NSSI

**Fig. 2:**
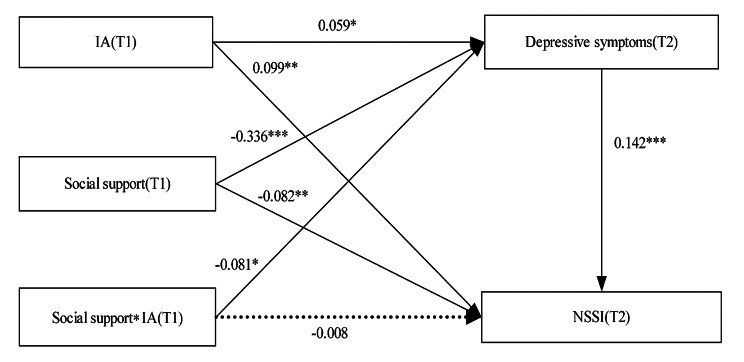
The moderating effect of social support on the indirect association between IA and NSSI mediated through depressive symptoms. Adjusted β coefficients are presented. The model adjusted for gender, age, ethnicity, regional areas, family structure, single child, family history of psychiatric disease, parenting style, monthly household income per capita, perceived academic pressure, education of main caregiver, baseline NSSI and baseline depressive symptoms. ^*^p < 0.05, ^**^p < 0.01, ^***^p < 0.001

**Fig. 3:**
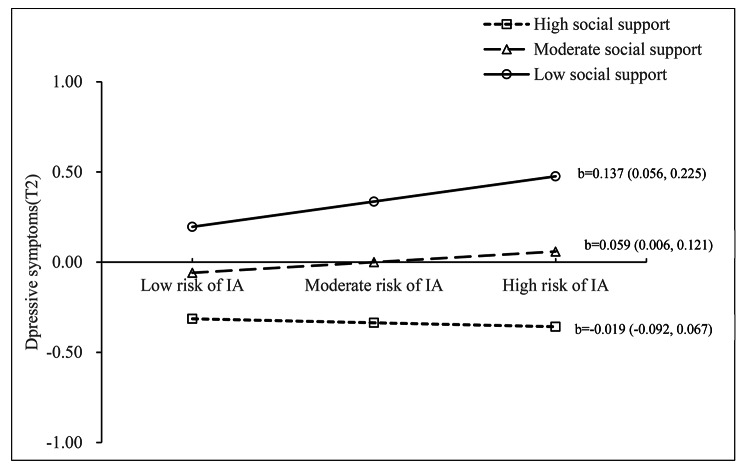
Interactive effect of IA(T1) and social support on depressive symptoms(T2)

The interaction between IA and social support at T1 in predicting depressive symptoms at T2 was significant (standard β= -0.081, 95%CI: -0.142 to -0.014), but was not significant in predicting NSSI at T2 (β=-0.082, 95%CI: -0.067 to 0.051) (Fig. 2, supplementary Table 4). The model of the SEM (Fig. 2) also fit the data well (CFI = 1, TLI = 1, RMSEA < 0.001, SRMR < 0.001). The simple slope test indicated that the association between IA at T1 and depressive symptoms at T2 was significant for participants with low (b = 0.137, 95%CI: 0.056–0.225) or moderate (b = 0.059, 95%CI: 0.006–0.121) level of social support, but not for those with high level of social support (b=-0.019, 95%CI: -0.092 to 0.067). Compared to participants with low level of social support, the positive links between IA at T1 and depressive symptoms at T2 was stronger for participants who had moderate social support (Fig. 3).

The indirect effect of IA on NSSI at T2 through depressive symptoms at T2 was conditioned at different levels of social support. Specifically, the indirect effect was strongest when social support was low (standard β = 0.019, 95%CI: 0.007–0.047), moderate at moderate levels of social support (standard β = 0.008, 95%CI: 0.001–0.025), and non-significant at high social support (standard β=-0.003, 95%CI: -0.015 to 0.009) (Supplementary Table 1). Similarly, the conditional indirect effect of IA on NSSI mediated through depressive symptoms was only found among females but not for males (Supplementary Table 5).

## Discussion

To our knowledge, this is the first longitudinal study examining the mediated effect of depressive symptoms and the moderating effect of social support in the association between IA and NSSI among adolescents. There were two key and novel findings from this study. Firstly, IA had a significant effect on NSSI at T2, 12.4% of which was mediated through depressive symptoms at T2. Secondly, social support moderated the effect of IA on NSSI at T2 through moderating the mediated effects of depressive symptoms. These findings provide new information about the association between IA and NSSI among adolescents, which could benefit educators, scholars and decision-makers to better understand the development of NSSI among children and adolescence and potentially inform NSSI interventions.

More and more social networking and learning are taking place online, especially during the COVID-19 pandemic. This could lead more people to depend on the internet, making them more susceptible to IA [30], especially among adolescents. Many studies have examined the potential effects of IA on adolescents’ mental and conduct difficulties, such as suicidal behaviors, NSSI, depression, anxiety, and attention deficit hyperactivity disorders [11, 31, 32]. In this study, we found that IA was significantly associated with an increased risk of NSSI at T2 among adolescents. This finding aligns with the only longitudinal study conducted in Taiwan [13] and most previous cross-sectional studies [11, 33], which support a positive association between IA and NSSI. However, two studies suggested a null association between IA and self-harm [34, 35], a broader term that includes NSSI but also encompasses suicidal behaviors. Inconsistencies across the collective findings may be due to different prevalence of NSSI and IA that were examined by different measurements and criteria, apart from study design and adjustments. Studies that have not distinguished between self-harm and NSSI may have overestimated the association between IA and NSSI [13, 36, 37]. Therefore, a more-uniform evaluative measurement of NSSI, apart from broader self-harm behavior, will be needed to clarify the association between IA and NSSI.

NSSI is often considered as an emotion-regulation strategy to decrease one’s emotional distress by distracting from intense emotion through the sight of blood, sensation of pain or focus on the injury itself [38]. Several studies have found the mediating role of depression between interpersonal stress and NSSI among adolescents, including peer bullying [18] and loneliness [39]. Although previous studies have indicated an association between IA and NSSI, as well as depressive symptoms with NSSI. However, there is a lack of study that explicitly addresses the mechanisms underlying IA, depressive symptoms and NSSI. Our study adds to this literature by demonstrating that depressive symptoms played a mediating role in the association between IA and NSSI. In other words, some adolescents with IA may not present with NSSI directly but instead present with depressive symptoms which then associates with an increased risk of NSSI. Indeed, our findings extend prior research by bridging the associations between IA, depressive symptoms and NSSI, which is in line with studies that have examined the indirect effects of IA on suicidal behaviors [40, 41]. Yu and colleagues conducted a cross-sectional study indicating that internet gaming disorders are positively associated with insomnia, which increases depressive symptoms, and, in turn, positively contributed to suicidal ideation [40]. Similarly, Guo and colleagues conducted a study of 20,895 adolescents, and found that sleep disturbance mediated the association between problematic internet use and suicidal behaviors [41].

The mediating role of depressive symptoms in the association between IA and NSSI could be explained in the several ways. Firstly, individuals with IA may be prone to be depression because they spend too much time in the internet virtual world of the internet and thus less time on social or group activities with family or peers, which can lead to unhelpful ways of adapting to their offline lives and increased isolation [17, 42]. Secondly, physiological studies have shown that IA could disrupt dopamine transmission by decreasing the expression of dopamine transporter in the striatum, which increases the risk of depressive symptoms [43]. The resultant depressive symptoms can, in turn, lead to NSSI as means to decrease emotional distress [44]. Therefore, interventions that focus on reducing depressive symptoms may be a potential strategy for preventing NSSI. Notably, the indirect effect of IA on NSSI mediated through depressive symptoms was significant in females but not in males, which may be related to the difference in psychological traits between females and males. Previous studies have reported that females experience higher rates of emotional disorders and tend to ruminate, avoid, and be less active in their coping methods, while males tend to be more impulsive and prefer to engage in physical and instrumental forms of comping methods directly [45].

Social support, defined as the extent to which individuals may receive emotional or instrumental help from others, is a significant predictor of adolescents’ positive psychosocial development [20]. High level of social support can protect against negative mental health outcomes, such as depression resulting from heightened life stress. For example, a cohort study of 1917 young adults examining associations between neighborhood-level social support and subsequent individual outcomes across 10 years, found that neighborhood-level social support can longitudinally protect against the onset of major depressive disorder in high-stress settings [20]. Similarly, the Avon Longitudinal Study of Parents and Children study also suggested that strong peer social support at age 15 may reduce the risk of depressive symptoms by the time children reach late adolescence [21]. Further, a study conducted in China suggested that social support had a moderating effect on the association between bullying and depressive symptoms [46]. Our findings build upon this literature by demonstrating that social support may mitigate the consequences of IA on depressive symptoms and the indirect effect of IA on NSSI through depressive symptoms.

A possible stress-buffering mechanism in this context may be that when individuals encounter a stressful event, adequate social support may mitigate the experience of stress and the onset of adverse outcomes by reducing or eliminating the stress reaction through calming the neuroendocrine system [19]. Specifically, social support has been found to buffer the effects of life stress on dopamine deficit, dysfunction, and diminish raised cortisol responses to social stressors [47, 48]. Contrary to our hypothesis, we did not find social support to buffer the direct effect of IA on NSSI. Previous studies have suggested that different sources of social support had a mixed stress-buffering effect. For instance, a study using data from the Adolescent Development of Emotions and Personality Traits found that only parental support, rather than peer support, protected adolescents from NSSI following a stressor [22], while another study conducted in China found that only friend support buffered the relationship between maltreatment and NSSI [49]. Therefore, it is possible that only specific sources of social support play a buffering role in term of the direct effect of IA on NSSI. This possibility warrants further research. Taken together, findings from the current study showed that social support serve as a protective factor, shielding adolescents from the detriment of IA, especially in female adolescents.

One major strength of our study is the sample’s representativeness. For this cohort study, we recruited a large sample size of participants in early adolescence from 3 cities that reflect the social, economic, and cultural status of China. Additionally, adjusting for a variety of potential confounders in the SEM and subsequent analyses ensured the validity and robustness of our findings. Moreover, this is the first study to examine the role of depressive symptoms and social support in association between IA and NSSI. This finding may be helpful to inform the development and implementation of preventive interventions aimed at addressing these concerns among adolescents, particularly for IA and NSSI.

### Strengths and Limitations

One major strength of our study is the sample’s representativeness. For this cohort study, we recruited a large sample size of participants in early adolescence from 3 cities that reflect the social, economic, and cultural status of China. Additionally, adjusting for a variety of potential confounders in the SEM and subsequent analyses ensured the validity and robustness of our findings. Moreover, this is the first study to examine the role of depressive symptoms and social support in association between IA and NSSI. This finding may be helpful to inform the development and implementation of preventive interventions aimed at addressing these concerns among adolescents, particularly for IA and NSSI. Several limitations should be noted. Firstly, our reliance on self-reported data from adolescents introduces the possibility of bias. Future studies should seek to obtain information from parents and other caregivers in addition to adolescents. Secondly, we did not differentiate between sources of social support (such as family support, peer support, community support), which prevented us from analyzing the moderating effect of different sources of social support on NSSI. Therefore, further study is needed to distinguish the moderating effect of different source of social support on NSSI. Thirdly, although our sample was representative, our study only included adolescents in grade seven. As such, we cannot generalize our findings to other study phases, as the prevalence of IA, depressive symptoms and NSSI, and source of social support may vary. Replicating our findings with other populations, and exploring the relationships we studied across other cultures would help to determine their generalizability. Hence, caution is required when applying our results to all adolescent populations.

## Conclusions

In this study, which involved a representative sample of seventh-grade adolescents, we observed the mediated effect of depressive symptoms and the moderating effect of social support in the association between IA and NSSI. The findings suggest that interventions aimed at reducing NSSI among adolescents should target IA and depressive symptoms, while also elevating social support.

## Electronic supplementary material

Below is the link to the electronic supplementary material.

Supplementary Material 1 Additional file: supplemental tables and figure

## Data Availability

The data that support the findings of this study are not openly available due to the intellectual property of the datasets belonging to the corresponding author and are available from the corresponding author upon reasonable request.

## Abbreviations

NSSI: Non-suicidal self-injury
IA: Internet addiction
IAT: Internet Addiction Test
CES-D: Center for Epidemiological Studies Depression Scale
SEM: structural equation models
COVID-19: Coronavirus disease 2019.
