# Supplementary material for: The role of depressive symptoms and social support in the association of internet addiction with non-suicidal self-injury among adolescents: a cohort study in China
Source: BMC Psychiatry. 2023 May 9;23:322. doi: 10.1186/s12888-023-04754-4 (PMC10169141; doi:10.1186/s12888-023-04754-4)
Supplement: Supplementary file 1 — Supplementary Material 1 Additional file: supplemental tables and figure [file 12888_2023_4754_MOESM1_ESM.docx]

**Supplementary Online Content**

**Supplementary Table 1.** Correlations among IA, depressive symptoms, social support and NSSI

**Supplementary Table 2.** Association between IA at T1 and NSSI at T2 by sex

**Supplementary Table 3.** Mediating effect of depressive symptoms between IA and NSSI by sex

**Supplementary Table 4.** Conditional indirect effect of IA on NSSI (T2) via depressive symptoms (T2)

**Supplementary Table 5.** Conditional indirect effect of IA on NSSI (T2) via depressive symptoms by sex

**Supplementary Figure 1.** Proposed model of association between IA, depressive symptoms, NSSI and social support.

**Supplementary Table 1.** Correlations among IA, depressive symptoms, social support and NSSI

| **Variables** | 1 | 2 | 3 | 4 | 5 | 6 |
| --- | --- | --- | --- | --- | --- | --- |
| 1. NSSI (T2) | 1 |  |  |  |  |  |
| 2. IA (T1) | 0.220^***^ | 1 |  |  |  |  |
| 3. Depressive symptoms (T2) | 0.274^***^ | 0.201^***^ | 1 |  |  |  |
| 4. Social support (T1) | -0.213^***^ | -0.173^***^ | -0.391^***^ | 1 |  |  |
| 5. NSSI (T1) | 0.276^***^ | 0.202^***^ | 0.188^***^ | -0.139^***^ | 1 |  |
| 6. Depressive symptoms (T1) | 0.295^***^ | 0.276^***^ | 0.295^***^ | -0.257^***^ | 0.389^***^ | 1 |

****p* < 0.001.

**Supplementary Table 2.** Association between IA at T1 and NSSI at T2 by sex

|  | **Males** | | |  | **Females** | | |
| --- | --- | --- | --- | --- | --- | --- | --- |
|  | **NSSI at T2 (N, %)** | **Adjusted ^a^**  **RR (95%CI)** | ***P*** |  | **NSSI at T2 (N, %)** | **Adjusted ^a^**  **RR (95%CI)** | ***P*** |
| No IA | 32 (5.7) | Ref=1 |  |  | 49 (10.8) | Ref=1 |  |
| Moderate IA | 47(17.6) | 2.34(1.78-3.78) | <0.001 |  | 50 (25.8) | 2.00(1.23-3.25) | 0.001 |
| Severe IA | 8(30.8) | 3.00(1.78-5.07) | <0.001 |  | 13 (46.4) | 2.44(1.93-2.91) | 0.001 |

a. Adjusted for sex, age, ethnicity, regional areas, family structure, single child, family history of psychiatric disease, parenting style, monthly household income per capita, perceived academic pressure, education of main caregiver, baseline NSSI and baseline depressive symptoms.

**Supplementary Table 3.** Mediated effect of depressive symptoms between IA and NSSI by sex ^a^

| **Variables** | **Males** | |  | **Females** | |
| --- | --- | --- | --- | --- | --- |
|  | **Standard β (95% CI)** | ***P*** |  | **Standard β (95% CI)** | ***P*** |
| IA (T1) → Depressive symptoms (T2) | 0.066(-0.012 to 0.146) | 0.105 |  | 0.103 (0.016 to 0.190) | 0.020 |
| Depressive symptoms (T2) → NSSI (T2) | 0.125(0.034 to 0.223) | 0.009 |  | 0.209(0.113 to 0.308) | <0.001 |
| IA(T1) → NSSI (T2) | 0.119(0.038 to 0.196) | 0.003 |  | 0.095(0.005 to 0.184) | 0.039 |
| Standardized effect |  |  |  |  |  |
| Indirect | 0.008(0.000 to 0.027) | 0.214 |  | 0.022(0.004 to 0.049) | 0.043 |
| Total | 0.128(0.048 to 0.202) | 0.001 |  | 0.116(0.026 to 0.206) | 0.012 |
| Mediating ratio (%) | 6.3(0.0 to 13.4) | -- |  | 19.0(15.4 to 23.8) | -- |

a. Adjusted with age, ethnicity, province, family types, single child, family history of psychiatric diseases, parenting style, monthly household income per capita, perceived academic pressure, education of main caregiver, baseline NSSI and baseline depressive symptoms.

**Supplementary Table 4.** Conditional indirect effect of IA on NSSI (T2) mediated through depressive symptoms (T2) ^a^

| **Variable** | **Consequence** | | | | |
| --- | --- | --- | --- | --- | --- |
|  | **Depressive symptoms(T2)** | |  | **NSSI(T2)** | |
|  | **Standard β (95% CI)** | ***P*** |  | **Standard β (95% CI)** | ***P*** |
| IA(T1) | 0.059(0.007 to 0.119) | 0.040 |  | 0.099(0.043 to 0.159) | 0.001 |
| Depressive symptoms(T2) | --- | --- |  | 0.142(0.071 to 0.214) | <0.001 |
| Social support(T1) | -0.336(-0.388 to -0.280) | <0.001 |  | -0.082(-0.133 to -0.028) | 0.003 |
| IA(T1) × Social support(T1) | -0.081(-0.142 to -0.014) | 0.013 |  | -0.008(-0.067 to 0.051) | 0.796 |
| NSSI(T1) | 0.050(-0.014 to 0.118) | 0.132 |  | 0.141(0.069 to 0.213) | <0.001 |
| Depressive symptoms(T1) | 0.153(0.083 to 0.222) | <0.001 |  | 0.122(0.046 to 0.197) | 0.002 |
| **Level of social support** | **Conditional indirect effect, Standard β (95% CI)** | | | | ***P*** |
| High level (+1SD) | -0.003 (-0.015 to 0.009) | | | | 0.658 |
| Moderate level (M) | 0.008 (0.001 to 0.025) | | | | 0.047 |
| Low level (-1SD) | 0.019 (0.007 to 0. 047) | | | | 0.021 |

a. Adjusted with age, ethnicity, province, family types, single child, family history of psychiatric diseases, parenting style, monthly household income per capita, perceived academic pressure, education of main caregiver, baseline NSSI and baseline depressive symptoms.

**Supplementary Table 5.** Conditional indirect effect of IA on NSSI (T2) mediated through depressive symptoms by sex ^a^

| **Sex** | **Level of social support** | **Conditional indirect effect, Standard** β (95% CI) | ***P*** |
| --- | --- | --- | --- |
|  | High level(+1SD) | 0.001 (-0.011 to 0.019) | 0.847 |
| Males | Moderate level(M) | 0.006 (-0.004 to 0.023) | 0.298 |
|  | Low level(-1SD) | 0.010 (-0.005 to 0.038) | 0.277 |
|  | High level(+1SD) | -0.017 (-0.043 to 0.002) | 0.087 |
| Females | Moderate level(M) | 0.007 (0.004 to 0.024) | 0.031 |
|  | Low level(-1SD) | 0.032 (0.001 to 0.071) | 0.028 |

a. Adjusted with age, ethnicity, province, family types, single child, family history of psychiatric diseases, parenting style, monthly household income per capita, perceived academic pressure, education of main caregiver, baseline NSSI and baseline depressive symptoms.


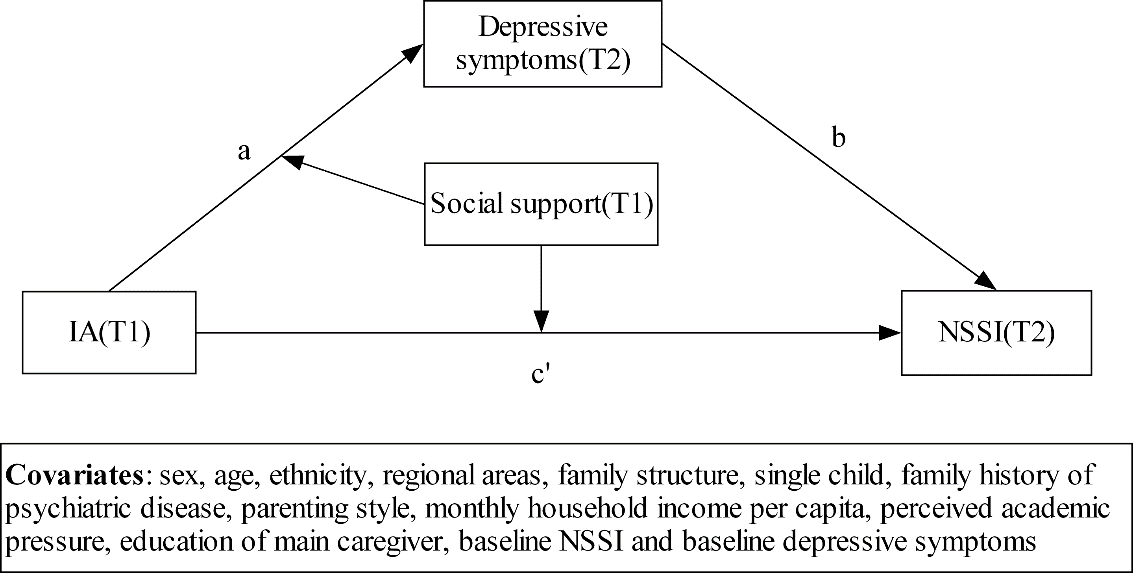


**Supplementary Figure 1.** Proposed model of association between IA, depressive symptoms, NSSI and social support.
